# Aerial Coverage Analysis of Cellular Systems at LTE and mmWave Frequencies Using 3D City Models

**DOI:** 10.3390/s18124311

**Published:** 2018-12-06

**Authors:** Achiel Colpaert, Evgenii Vinogradov, Sofie Pollin

**Affiliations:** Department of Electrical Engineering, KU Leuven, 3000 Leuven, Belgium; evgenii.vinogradov@kuleuven.be (E.V.); sofie.pollin@kuleuven.be (S.P.)

**Keywords:** UAV, LTE, mmWave, beamforming, cellular, drone, interference, SINR

## Abstract

Cellular connectivity for UAV systems is interesting because it promises coverage in beyond visual line of sight scenarios. Inter-cell interference has been shown to be the main limiting factor at high altitudes. Using a realistic 3D simulator model, with real base station locations, this study confirms that UAVs at high altitudes suffer from significant interference, resulting in a worse coverage compared to ground users. When replacing the existing base stations by mmWave cells, our results indicate that ground coverage is decreased to only 90%, while UAVs just above rooftop level have a coverage probability of 100%. However, UAVs at higher altitude still suffer from excessive interference. Beamforming has the potential to improve mmWave link budget and to decrease interference and is for this reason a promising technology for ensuring connectivity to aerial users.

## 1. Introduction

Unmanned aerial vehicles (UAVs) are getting more common and important in everyday life, but regulation typically constrains the use of UAVs to visual line of sight situations [1,2]. The most important UAV applications, including search and rescue, packet delivery or autonomous surveillance, would require beyond visual line of sight operation. Inclusion of UAVs in Long-Term Evolution (LTE) networks has received significant interest in recent years, as this promises reliable coverage and good throughput, required for the beyond visual line of sight applications. Using cellular networks to provide connectivity to the UAVs introduces new challenges, for instance the resource sharing between aerial and ground users becomes a problem since the radio environment at high altitudes differs from the ground-level one.

The analysis by Qualcomm in [3] is based on a large-scale measurement campaign and supports the viability of LTE commercial mobile networks for UAV operating beyond visual line of sight. In the report, it is shown that the signal quality of the downlink, from Base Station (BS) to UAV, was significantly lower for flying UAVs compared to the ground users: Signal-to-Interference-and-Noise (SINR) levels decrease up to 5 dB due to inter-cell interference. However, the SINR did not decrease to an extent that the UAVs had no coverage (defined as SINR ≤ −6 dB). The coverage outage probability was found to be very similar for UAVs and ground user equipment (UE) devices and it was concluded that commercial LTE networks should be able to support downlink communications requirements of the LTE-connected UAV deployment without modifications.

In [4], the authors studied the feasibility of wireless connectivity for UAV user equipment via LTE networks. Moreover, propagation characteristics of base station to UAV communications were studied using measurements and ray tracing simulations in a rural area. They concluded that, for high altitudes propagation, conditions are close to free space and that at high altitudes downlink connections suffer from higher interference due to higher probability of LOS propagation of interfering base stations.

Additionally, Van Den Bergh et al. [5] analyzed the impact of UAVs on the performance of an LTE network through simulations and measurements. They considered two scenarios, one where UAVs act as base stations, providing connectivity to ground users, and another scenario where UAVs act as users, connecting to a ground network. They studied the UAVs’ impact on downlink and uplink performance of the ground LTE network. They concluded that interference would be the main limiting factor when introducing LTE enabled UAVs.

The authors of [6] performed simulations and field measurements with a UAV in a rural area, evaluating the performance of aerial radio connectivity. They concluded that interference was one of the main limiting factors for both downlink and uplink connections, especially when load was high in the network. Two antenna mitigation techniques were explored. First, antenna beam selection was considered, where the UAV selected a directive antenna based on the location of its serving base station, and secondly interference cancellation was investigated, exploiting techniques to remove interference at the receiver. Alternatively a novel downlink inter-cell interference coordination mechanism was introduced in an effort to improve overall system performance and reliability, both for aerial and ground users.

The measurements and simulations of [3,4,5,6] show that interference was found to be the main limiting factor for different environments, in line with theoretical studies. However, the optimistic quality estimations presented in [3] could not be confirmed by Lin et al. [4], Nguyen et al. [6] and Van Den Bergh et al. [5]. Note that these works [3,4,5,6] considered only rural and coast scenarios.

The analysis in [7] describes the coexistence of aerial and ground users in cellular networks. The authors proposed a downlink coverage performance characterization framework to optimize a cellular network serving both aerial user equipment (AUE) and terrestrial user equipment. This framework considers the impact of fundamental design parameters such as base station height, antenna pattern and UAV altitude for different types of environments. They found that an optimal UAV altitude with respect to coverage probability does exist. Follow-up research in [8] analyzed the performance of a cellular network using closed form expressions and found network design trade-offs to optimize the cellular network for both ground users and UAVs. Their methods however are purely analytical and do not allow estimating performance of an existing network in a real scenario.

Several approaches to overcome the interference problem are suggested in the literature. The work in [9] developed an interference-aware path planning scheme for aerial UEs that yields to a minimum communication latency for UAVs as well as their interference on ground users. In [10], it was proposed to transfer information over a satellite link as an addition to the cellular connection. Some other techniques attract attention of the research community: Massive Multiple Input Multiple Output (MaMIMO) and analog beamforming could be major technologies when trying to include UAVs in future cellular networks. Moreover, in upcoming 5G networks, millimeter wave (mmWave) cellular networks will play a big role in providing higher data rates and even more expanded coverage [11]. It is well known that mmWave links are much more vulnerable to blockages; however, it has been shown in all mentioned papers that UAVs have higher probability to have no obstacles between aerial and ground terminals.

The other known problem of propagation at high frequencies is high attenuation of the signal, which may be overcome by using advanced antenna techniques giving large array gains. The combination of the beamforming and mmWave for UAV applications was analyzed in [12]. The small antenna aperture size of mmWave systems allows the deployment of large antenna arrays. These large arrays support the usage of analog and digital beamforming and MaMIMO techniques. These techniques allow more targeted use of the spectrum, effectively serving multiple users without creating interference to their neighbors, resulting in mmWave cellular systems being noise limited rather than interference limited unlike LTE cellular systems [13,14]. Potentially, the combination of these technologies will be able provide a solution to the congestion of the spectrum for UAVs.

Published research considers rural environments for measurements. The simulation based works rely on modeling of various Manhattan-like urban areas or on stochastic channel and LOS probability models. We believe that more realistic urban models must be included in the analysis since the propagation conditions are highly dependent on the environment. Moreover, using UAVs in a realistic European middle-sized city has not been studied. Initial studies of mmWave communication with UAVs have not answered several important questions:

- From what altitude onwards can we assume LOS communication in an urban environment?
- Is mmWave communication superior to LTE for providing coverage to UAVs, as it enables networks with less interference?
- Can we have a good aerial coverage at mmWave frequencies using the same base station density (as claimed in [15]), or is it necessary to deploy many more cells?
- As mmWave promises cells with more directive antennas, how directional should the antenna array be for optimizing aerial coverage?

In this article, we investigate these key challenges in mmWave UAV-enabled cellular networks and discuss possible solutions. To answer the questions above, we perform simulations in a 3D environment of real world locations. In the paper, this environment is statistically characterized and we discuss the possible mmWave semi-deterministic channel models. The reference models of antenna patterns complimented with real base station positions are considered in the analysis. Next, we provide a detailed performance comparison of the LTE and mmWave cellular networks. We present a method to improve the interference situation for mmWave networks by tuning the number of sectors per BS as a basic form of beamforming.

The remainder of this paper is organized as follows. Section 2 introduces the system model and the semi-deterministic channel model as well as the description of our simulator and its key parameters. The simulation results for LTE and mmWave cellular networks (with and without interference mitigation applied) are presented in Section 3. Section 4 concludes the paper.

## 2. System Model

This section describes the system model that is used to simulate the viability and performance of UAVs in existing LTE cellular networks as well as UAVs in a simulated mmWave cellular network. For LTE, the simulations use the 1.8 GHz frequency band. Out of all 5G NR mmWave operating bands specified in [16], band n260 is chosen, specifying a frequency band of 37–40 GHz. Within this band, an operating frequency of 38 GHz was chosen, as for this frequency there is already extensive research on urban models and parameters [17,18,19]. The spectrum at 38 GHz is less congested and allows for a higher bandwidth *B*, however a higher bandwidth also introduces more noise. In this article, we consider B=20 MHz and B=400 MHz; by doing this, the effect introduced by using a higher carrier frequency could be separated from the effect of using a higher bandwidth. The simulations focus on the downlink connection (i.e., from BS to UAV) of UAVs flying up to an altitude of 250 m above ground level (AGL). The research focuses on the interference experienced by the UAV.

### 2.1. Network Model

To study the influence of interference on the network performance for UAVs, a downlink simulator combining multi-band channel models with a 3D map of a real city was designed. Table 1 contains the main simulation parameters.

#### 2.1.1. Environment

**Figure 1:**
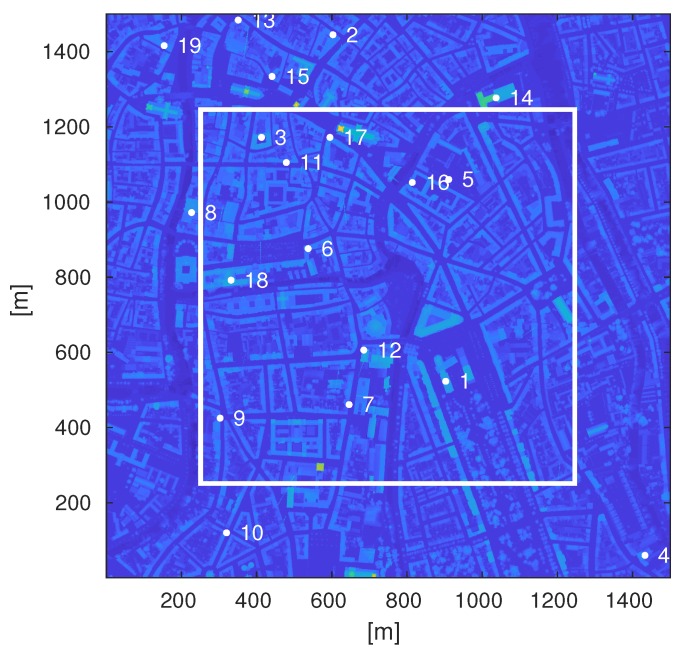
Surface map of the city of Ghent with region of interest marked by the white square and the locations of the considered base stations marked by the white dots. The numbers indicate the index of the base stations.

All simulations were performed in a real 3D environment. The 3D surface scan with 1 m resolution of Flanders, Belgium [21] was used. It is a height map containing buildings as well as vegetation (see Figure 1). The city of Ghent was chosen for the simulations, since it represents a typical European middle-sized city. The environment can be categorized as urban.

The simulator uses real locations of the base stations provided by BIPT [22]. Nineteen BS in a radius of 750 m around the center of the map were considered. To imitate the mast deployment, the height of the BS was determined by the highest location in a radius of 5 m around the given BS coordinate to guarantee that it is on top of a roof and not in the middle of a street. It was put at 5 m above this point make sure it can be seen at street level, even if put on top of a structure. The BS heights ranged from approximately 23 m to 32 m, with the average height being 27.81 m. In the baseline LTE scenario, every base station was considered to have three sectors, while, for the mmWave scenarios, the number of sectors was increased up to 45 sectors per base station (see Section 2.2). We considered the infrastructure of a single operator. No microcells were considered.

Figure 1 shows the map with the region of interest marked by the white lines and the base station locations marked as white dots. An area of 1 km${}^{2}$ (see Figure 1) centered at 51°2′57″ N 3°43′41″ E is used for the analysis.

#### 2.1.2. Link Model

This subsection explains all the components out of which the link model exists.

*Antenna patterns*: For both considered frequency bands, the BSs were equipped with antennas with 3D radiation patterns modeled as in [23], as it specifies two different models for sectoral antennas, one for below 3 GHz and one for from 3 GHz to 70 GHz. An electrical downtilt of eight degrees and no mechanical tilt was used throughout all simulations. The 3 dB beamwidth ($\phi_{3}$), maximum transmit power ($P_{tx}$) and maximum antenna gain ($G_{0}$) for the three-sector case were based on the values provided by BIPT [22] (values can be found in Table 2). For mmWave deployments, it was assumed that more directional antennas, hence deployments with more than three sectors, can be designed. For the >3-sector cases, the values of $\phi_{3}$ are derived from the three-sector case by proportionally dividing the 65${}^{\circ }$ beamwidth. Using the formulas of ITU-R [23], the new maximum antenna gain, $G_{0}$, could be derived for every case and the maximum transmit power, $P_{tx}$, was adjusted proportionally to the new maximum gain as to keep the exposure for ground users constant, in line with current RF exposure regulatory constraints. Reducing the azimuth beamwidth of the antenna patterns increases the maximum gain, which results in the maximum transmit power gradually lowering with increasing number of sectors per base station, the goal is to keep the sum of the maximum gain and the maximum transmit power constant. The values of $\phi_{3}$, $P_{tx}$ and $G_{0}$ for different number of sectors can be found in Table 2. As for the azimuth rotation of every sector, the first sector was pointed north, 0${}^{\circ }$, and the other sectors were evenly spaced to other angles. For example, in the six-sector case, the angles of the sectors were [0${}^{\circ }$, 60${}^{\circ }$, 120${}^{\circ }$, 180${}^{\circ }$, 240${}^{\circ }$, 300${}^{\circ }$]. For both ground user equipment (UE) and aerial user equipment (AUE), an omnidirectional antenna with maximum gain of 2.15 dBi was used.

**Figure 2:**
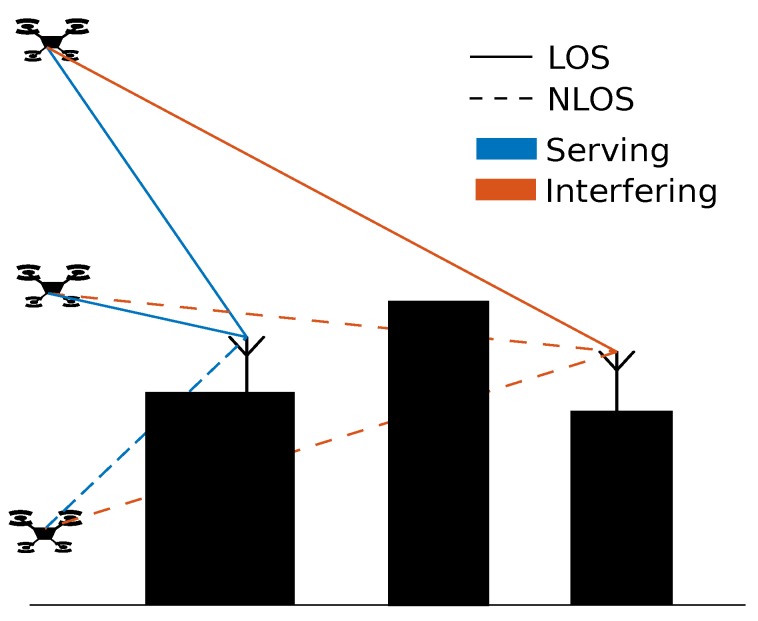
A 2D representation of the line of sight. Blue lines indicate the serving base station, while orange lines indicate interfering base stations. A dashed line indicates NLOS and a full line indicates LOS.

*Line of sight*: When checking whether a UE location is in LOS with a base station, a line is drawn between the base station and the user in the 3D environment. If there are no intersections with the 3D environment the link between user and base station is considered LOS and if obstructions do exist the link is considered non-line of sight (NLOS). A 2D representation can be seen in Figure 2.

*Path loss model*: The path loss (PL) model for LTE is defined by combining the work in [24], which specifies an LTE urban macro cell path loss model for ground users up to 22.5 m AGL, and the work in [25], which defines an LTE urban macro cell path loss model for aerial users above 22.5 m AGL. The general expression is as follows:

(1) $$PL=C+D\times log_{10}\left(d\right)+20log_{10}\left(f_{c}\right)+X_{\sigma },$$

where *C* and *D* are scenario (i.e., environment and LOS/NLOS link) and altitude-dependent constants (for the values, refer to 3GPP [24,25]); d is the 3D distance to the basestation; $f_{c}$ is the carrier frequency, in this case 1.8 GHz; and $X_{\sigma }$ is a zero mean Gaussian random variable with standard deviation σ, which is determined by UE altitude. For example, the formula for a user at an altitude above 22.5 m and with a LOS connection is defined as follows:

(2) $$PL=28.0+22\times log_{10}\left(d\right)+20log_{10}\left(f_{c}\right)+X_{\sigma },$$

where σ is defined as follows:

(3) $$\sigma =4.64\times exp(-0.0066\times h_{ut}),$$

where $h_{ut}$ is the UE altitude. To calculate the average building height, which the ground user model uses as an input [24], a line is drawn between user en BS and the average building height of all buildings intersecting this line is calculated.

For mmWave, we consider the following close-in reference distance path loss model of [18]:

(4) $$PL\left(d\right)=PL\left(d_{0}\right)+10nlog_{10}\left(\frac{d}{d_{0}}\right)+X_{\sigma },$$

where $X_{\sigma }$ is a zero mean Gaussian random variable with standard deviation σ and *n* is the path loss exponent for a particular frequency band or a given environment. The standard deviation values for the LOS and NLOS cases are 10.3 and 14.6, respectively [17]. The path loss exponent values for the LOS and NLOS cases are 2.20 and 3.88, respectively [17], and $PL(d_{0})$, the free space reference path loss at distance $d_{0}$, is defined as:

(5) $$PL\left(d_{0}\right)=10log_{10}{\left(\frac{4\pi d_{0}}{\lambda }\right)}^{2},$$

where λ is the wavelength and $d_{0}$ is the reference distance. The reference path loss is taken at a distance of 1 m as proposed in [19] and thus results in 64 dB reference path loss.

*SINR calculation*: When calculating the SINR at a specific location, all BSs are assumed to be transmitting on all sectors. Thus, for every sector, the received power is calculated as follows:

(6) $$P_{rx}=P_{tx}+G_{tx}+G_{rx}-PL,$$

where $P_{rx}$ is the received power, $P_{tx}$ is the constant transmitted power, $G_{tx}$ and $G_{rx}$ are, respectively, the transmitting and receiving antenna gains in the direction of the user and PL is the corresponding path loss for that location, with all parameters in dB.

Let A be the collection of all sectors. For one specific location, the SINRs of all sectors are calculated as follows:

(7) $$\forall a\in A:SINR_{a}=\frac{P_{rx,a}}{I_{a}+N},$$

where $N=N_{0}BF$ ($N_{0}$ is white noise density, *B* is signal bandwidth and *F* is noise figure, values in Table 1) is the noise power and $I_{a}$ is the interference power considering *a* is the serving sector, which is defined as follows:

(8) $$I_{a}=\sum_{i\neq a}^{A}P_{rx,i}.$$

**Figure 3:**
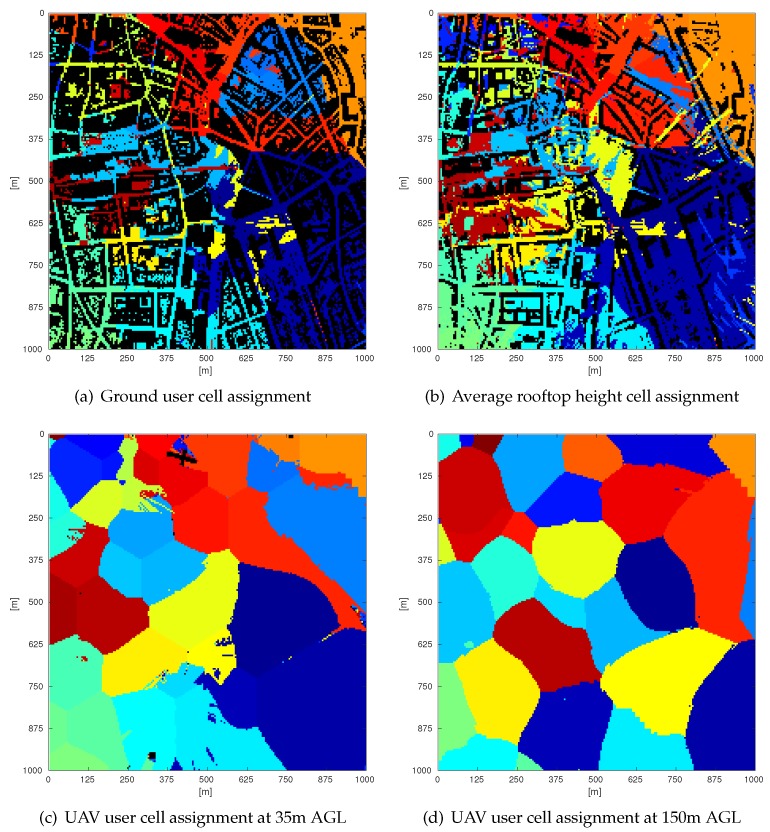
Cell assignment at different altitudes in a three-sector scenario. Each unique color corresponds to a unique sector. A pixel with a specific color indicates to which sector a user at this specific location will be assigned. A black pixel indicates a building.

Out of all the resulting SINR values, the highest value is chosen as serving sector for every location, which allows us to determine a 3D sector assignment map that shows the sector assignment in a geographical area. Four slices of this assignment map at different heights are shown in Figure 3. Figure 3a–c shows that up to an altitude of around 35 m assignments happen in a cell-like manner, which means a UE is most likely to be assigned to the most nearby base station. However, looking at Figure 3d, it can be seen that at an altitude of 150 m an AUE is not necessarily assigned to the closest BS due to the shape of the 3D antenna radiation patterns and the AUE is not in the main lobe of the radiation pattern.

### 2.2. Scenarios

Below, a description of all parts of the simulation cases is given.

#### 2.2.1. Load Types

Two different load cases were considered. The first case is the operation of base stations in full-load. This is the worst case scenario for UAVs, as indicated by Nguyen et al. [26], because all neighboring cells have data to transmit in downlink and thus cause inter-cell interference to the receiver, especially if this receiver is a UAV at a high altitude. In the simulations, all base stations were made to transmit simultaneously on all sectors with their respective power $P_{tx}$ (see Table 2).

The other case is operation in partial load. In partial load operation, active ground users are distributed randomly in the city with a certain density, ρ, using a Poisson Point Process. The sectors that contain an active user, according to the assignment map, are turned on and are behaving as possible interferers. Not every sector is on and this results in an increase in SINR. In the partial load case, interference power can be calculated as follows:

(9) $$I_{a}=\sum_{i\neq a}^{A}P_{rx,i}P\left(A_{i},\rho \right),$$

where $P(A_{i},\rho )$ is the probability that a base station is transmitting on a certain sector depending on the ground surface area of that sector $A_{i}$ and the active user density ρ, with $P_{rx,i}$ being the power that is received when the sector is transmitting. Several simulation runs were performed with new user locations each run. Values for ρ and the number of iterations can be found in Table 1.

**Figure 4:**
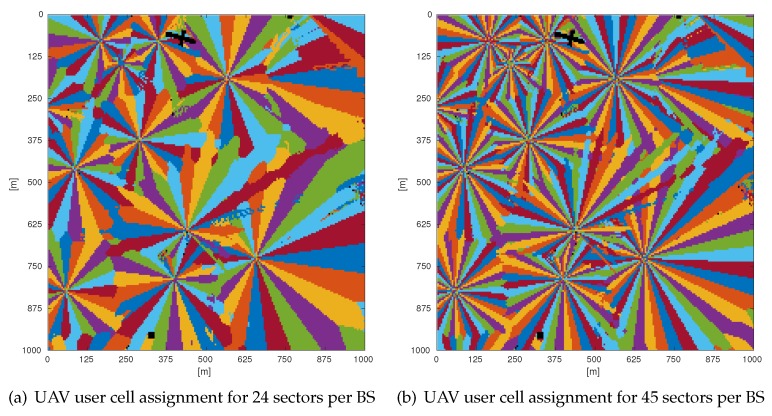
Examples of cell assignment in the case of 24 and 45 sectors per base station at an altitude of 35 m AGL.

Following the partial load case, we tried to reduce the interference power by increasing the number of sectors per base station to create narrower sectors and thus smaller sector areas. This can be achieved by deploying phased antenna arrays, which is an easy way to reduce the azimuth beamwidth of an antenna radiation pattern. A reduced ground area of a sector reduces the probability that the base station will transmit in this sector. In our simulations, when increasing the number of sectors, all antenna parameters are scaled proportionally, as explained in Section 2.1.2 (parameters can be found in Table 2). Note that a high number of sectors (45) results in a very small 3 dB beamwidth (4.33${}^{\circ }$); we assume this is possible using phased antenna arrays at mmWave frequency. Figure 4a,b shows example scenarios where the number of sectors per base station is increased to 24 and 45, respectively.

#### 2.2.2. Coverage Probability

Coverage for a specific location in 3D space is calculated as true or false depending on whether the SINR at that given location is above a certain threshold. The performance of every link was estimated using the coverage probability at several altitudes, which is calculated by dividing the number of locations at a certain altitude where there exists coverage by the total number of considered locations at that altitude. Coverage probability represents how likely a UAV at a certain altitude will have a reliable link to any BS. The target SINR depends on the throughput requirement. UAVs could only need a command and control downlink, which results in an estimated data rate of only 60–100 kbps for the downlink [27]. As stated in [27], this requires a minimum SINR value of −6 dB. By focusing on the lowest possible rate, a high coverage probability and hence best possible reliability is achieved.

## 3. Simulation Results

### 3.1. Environment Characterization

First, some characterization and analysis of the environment was performed. Basic parameters such as building height and line of sight were calculated.

**Figure 5:**
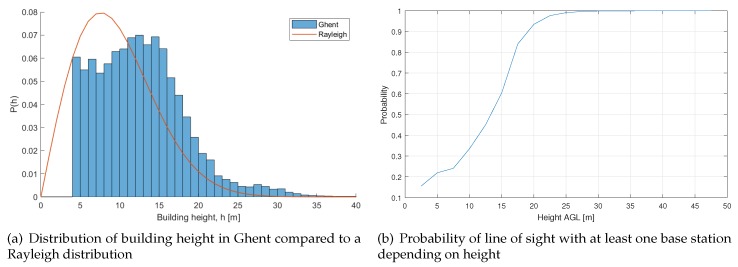
Statistics extracted from the 3D map.

A comparison between the resulting building height distribution and a fitting to the Rayleigh distribution suggested in [28] can be seen in Figure 5a. We found a minimum building height of 4 m. As can be seen, the distribution overestimates the amount of buildings with a height up to 10 m and underestimates the amount of buildings with a height larger than 10 m. The calculated mean building height is 13.32 m, which was calculated with respect to the base area of every building.

A probability of line of sight was also calculated to characterize the environment. The probability of line of sight is presented as a function of UE height and indicates whether the user is in LOS with any base station. When determining whether a ground or aerial user is in LOS with a base station, the 3D map is used, as explained in Section 2.1.2. The resulting LOS probability can be seen in Figure 5b. In our example, the simulations showed that a UAV is always in LOS with at least one BS if the UAV flies higher than 30 m AGL.

### 3.2. LTE vs. mmWave

Given our 3D propagation model, it is possible to realistically compare coverage performance of LTE with 5G or mmWave. For this simulation, full-load and the three sector case is considered, and it is assumed that the LTE and mmWave BSs all have the same location. First, the SINR curves are generated, followed by the coverage probability curves.

**Figure 6:**
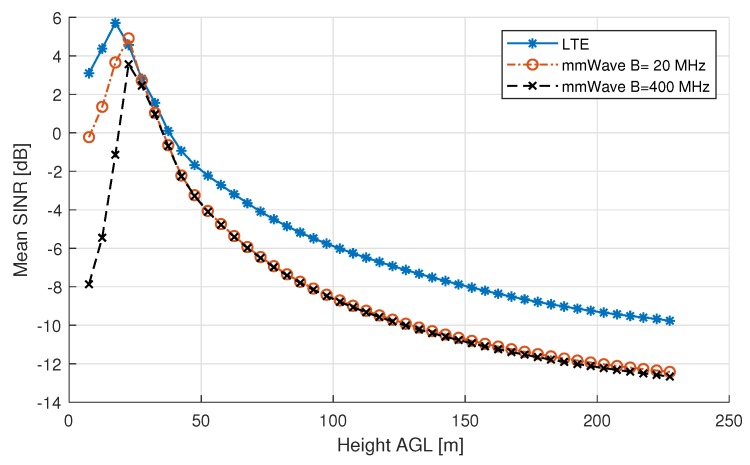
Mean SINR as a function of height AGL.

The results of the simulation, as shown in Figure 6, indicate that SINR is significantly decreased (with respect to the ground level) for high UAV altitudes, because of interference.

For ground users, LTE at 1.8 GHz outperforms the mmWave band by approximately 4 dB on mean SINR if the same bandwidth of 20 MHz is considered. This difference is due to the fact that, for mmWave, users having a NLOS link receive almost zero power ($P_{rx}\approx 0$) from their serving base station, thus the received power is below the noise floor, resulting in a negative SINR, even when the interfering signals are blocked. For an increased bandwidth of 400 MHz, the SINR levels at the ground level are even lower due to the increase in noise power, while the serving and interfering signal’s power stay the same.

Right above rooftop height (13.32 m), we can observe a peak in SINR for both LTE and mmWave. Both peaks can be explained by the fact that a user will be likely to get a LOS connection with its serving base station, while all interfering BSs, which are at a larger distance of the user, will still be in NLOS. The existence of both peaks is in line with multiple research contributions dedicated to optimal positioning of an AUE [8,29]. The maximum mean SINR of the mmWave system occurs at a slightly higher altitude.

For high altitudes, SINR decreases for both LTE and mmWave as more interfering base stations will have a LOS link. Interestingly, increasing the bandwidth does not affect mean SINR at high altitudes, meaning that at high altitude interference becomes the major limiting factor for both considered carrier frequencies, regardless of the bandwidth used. For high altitudes (>50 m), the difference in SINR of both frequencies is constant. This difference can be attributed to the frequency difference which results in a fixed difference in PL, which behaves as a free space path loss at these altitudes.

**Figure 7:**
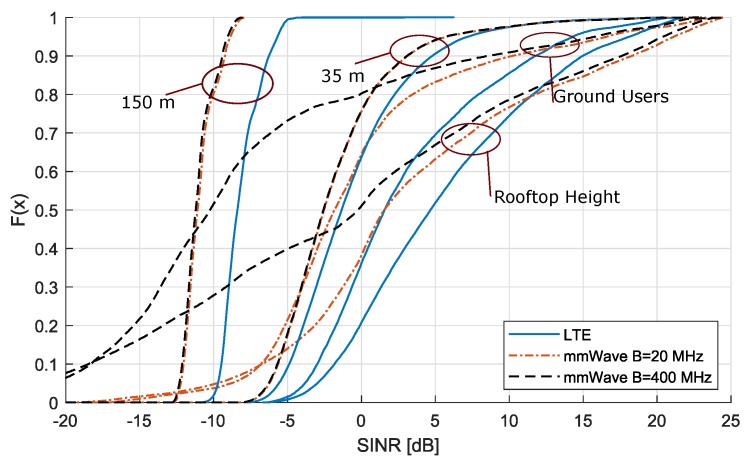
Cumulative distribution of SINR in full-load at different heights comparing LTE and mmWave.

Figure 7 shows the cumulative distribution function of SINR in function of altitude. For one specific altitude, all SINR values are considered and plotted as a distribution, for both LTE and mmWave scenarios. We can see that, for altitudes up to right above rooftop, the SINR spread is limited to a range of −5 dB to +20 dB for B=20 MHz.

Increasing the bandwidth results in a larger spread for the altitudes below the rooftop; however, when interference becomes high due to higher LOS probability, SINR distributions for both bandwidths behave identically. Moreover, at the altitude of 35 m, the distribution for LTE and mmWave is more or less the same. For high altitudes, the SINR is almost constant and there exists a fixed difference between LTE and mmWave SINR, as explained in previous paragraph.

**Figure 8:**
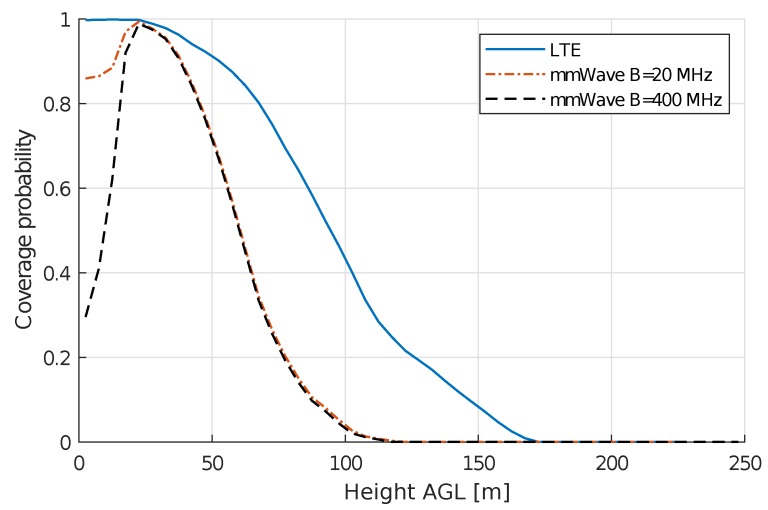
Coverage probability for specific heights in a full-load scenario, representing the probability of having an SINR of −6 dB or higher at a certain altitude.

When looking at the results of coverage probability, LTE has better terrestrial coverage due to the lower PL in this frequency band (see Figure 8). For the full-load case, we notice that, for ground users in the mmWave scenarios, the coverage probability is not 100% while for LTE it is, as expected from our simulations. The low coverage probability can be explained by the lower SINR values for ground users using mmWave as the results in Figure 7 show, especially for the 400 MHz bandwidth scenario almost 40 % of the spread is located below −6 dB, while for LTE the complete spread is located above −6 dB. After reaching its peak at a height of 22 m AGL, which is above the average rooftop height, mmWave coverage probability drops faster than LTE’s and reaches zero at an altitude approximately 50 m lower than LTE. These results suggest that mmWave is a poor choice for aerial vehicles in a full-load scenario. However, mmWave enables usage of more advanced techniques such as beamforming and MaMIMO due to the use of large antenna arrays. These techniques can be used to decrease interference, especially for AUE.

We can conclude that interference is still the main limiting factor for UAVs deployed in a cellular system, even when using mmWave, as described in the literature [4,5,6,7,26]. The result of an optimal altitude for UAVs right above rooftop height is in line with existing research [7,8,29]; however, our results show a higher coverage probability for ground users. This difference can be explained by the higher SINR threshold used in those papers. We found different coverage probabilities for UAVs and ground users, contrary to the authors of [3] who found a similar coverage probability for both ground and aerial users. This inconsistency can be a result of the fact that they performed experiments in a semi-urban environment with a lower density of base stations. Moreover, the fact that we assumed a worst-case network in full-load would also result in more interference.

### 3.3. Interference Management

In the previous section, we present the analyses of a system operating in full-load, i.e. all base stations were simultaneously transmitting data on all three of their sectors. However, in reality, most of the time base stations would only transmit on a certain sector when communication is requested by an active user in that sector’s area. In the next simulation, we assumed a partial load and we increased the number of sectors per base station, as explained in Section 2.2, only for the mmWave frequency band. Increasing the number of sectors and keeping the active ground user density ρ (Table 1) constant would decrease the probability that an interfering base station, with sectors oriented towards an AUE location, is transmitting on those sectors, thus decreasing the amount of interference and increasing the SINR for that specific AUE location. Note that the maximum transmit power is decreased with increasing number of sectors due to RF exposure regulations (as explained in Section 2.1.2), thus not resulting in a better link budget.

**Figure 9:**
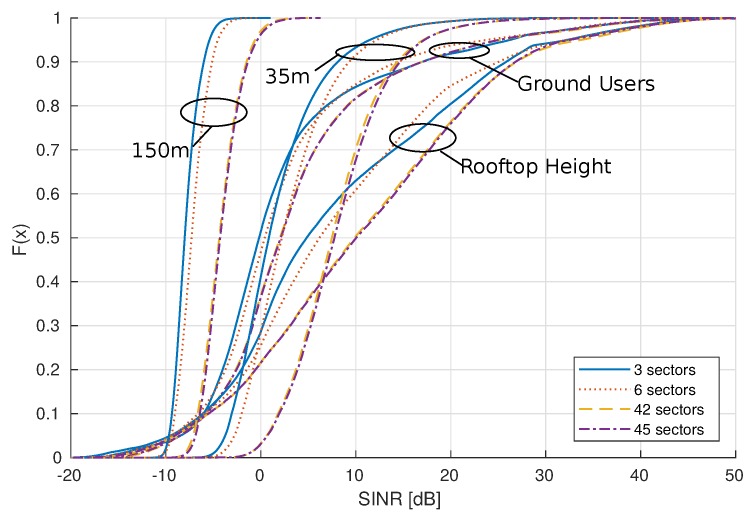
Cumulative distribution of SINR in partial load at different AUE altitudes comparing different numbers of sectors per base station for the mmWave scenario.

Figure 9 shows the SINR distributions at different heights when varying the number of sectors per base station. Note that the results for the 20 MHz bandwidth are demonstrated here and only for the mmWave scenario. Increasing the number of sectors improves the SINR values. For ground users, the increase in SINR is negligible, while, for AUEs at high altitudes, it is approximately 5 dB when increasing the number of sectors from 3 to 45.

**Figure 10:**
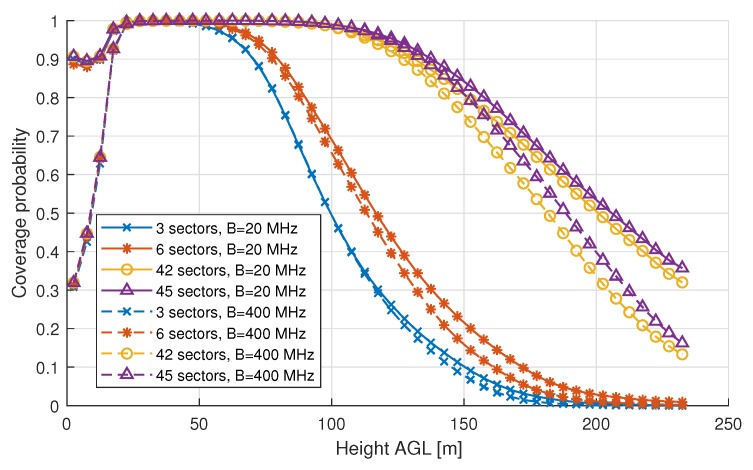
Coverage probability for specific heights in a partial load scenario (mmWave only), representing the probability of having an SINR of −6 dB or higher at a certain altitude. Different symbols represent different number of sectors per base station.

When looking at the results of coverage probability (see Figure 10), we can conclude that a significant improvement can be achieved when using a high number of sectors. For example when using 45 sectors, a coverage probability of 90% is present up to approximately 140 m AGL, which is a reasonable maximum height for UAV deployment. Figure 10 shows that increasing the bandwidth to 400 MHz does not have a significant effect for high altitudes, which indicates that AUE are in fact still interference limited instead of being noise limited, even when using mmWave communications. Nonetheless, the statement of noise limited mmWave communications does still hold for ground users up to rooftop height.

**Figure 11:**
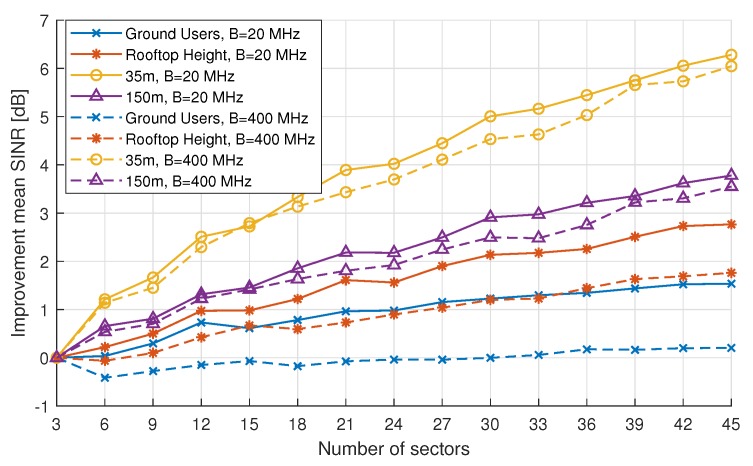
Improvement of mean SINR in partial load over the three-sector case at different heights for a mmWave network.

Improvements however saturate, as Figure 11 indicates, and, at some point, increasing the number of sectors will only give a minimal increase in SINR and will not outweigh the extra cost of more expensive mmWave equipment. Figure 11 also gives a clear indication at which altitudes the mmWave system is noise-limited and at which altitudes it is interference-limited. The lines indicating the increase in SINR at altitudes of 35 m and 150 m at 20 MHz are almost equal to their 400 MHz counterparts, indicating that at these altitudes an increase in bandwidth, resulting in an increase in noise power, does not significantly affect the SINR. One can conclude that at these altitudes the mmWave system is indeed interference limited. When looking at the lines for ground and rooftop users, one can see that there exists a clear difference between the 20 MHz and 400 MHz line, especially for ground users; it is clear that, when considering a large bandwidth, the increase of number of sectors does not result in any improvement of the mean SINR. This result is a clear indication that increasing the bandwidth and thus the noise does affect the SINR at low altitudes, resulting in a noise-limited system for ground users. These results indicate that, when deploying a mmWave system for AUEs, interference is still the main problem and bandwidth is not a problem. However, when deploying a mmWave system for ground UEs, blockage is still the main problem and more care should be taken when deploying base stations.

## 4. Conclusions and Further Work

In this article, we estimated the performance of LTE and future mmWave cellular networks for serving flying UAVs using realistic 3D maps of a typical European middle-sized city and semi-deterministic channel models. Using a mmWave network deployed in the same manner as the current LTE cellular network, a comparison between LTE and mmWave was made and the results showed that inter-cell interference remains the main problem for ground to air communication at high altitudes, as described in the existing literature [4,5,6,7,26]. Contrary to Qualcomm [3], our results indicate that deploying UAVs at high altitudes will result in their coverage probability dropping below 90% at altitudes above 50 m AGL, when using the existing LTE cellular network.

The results showed that an optimal deployment altitude for AEUs does exist, as analytically derived in [7], both for an LTE network and a mmWave network, albeit not at the same altitude due to different blockage effects at these frequencies. For a full-load scenario, LTE outperforms a mmWave cellular network at providing coverage at high altitudes when using the same BS locations. Using a mmWave communication link, ground UEs experience too much blockage by buildings to have good coverage. AUEs at high altitudes have a lower SINR using a simulated mmWave system than when they would using the existing LTE system. The results show that mmWave reaches a coverage probability of 0% at an altitude approximately 50 m lower than LTE.

When operating in high-load, both an LTE and a mmWave system fail to provide coverage of to AUEs at altitudes higher than 50 m. However, for a partial load scenario, increasing of the SINR levels at higher altitude is possible when a mmWave system is used, especially when the number of sectors per base station is increased, using mmWave phased antenna array techniques. For mmWave networks, the mean SINR level at higher altitudes can be improved by 1–7 dB compared to the standard three-sector case. The largest improvement of SINR can be seen around the height of 35 m. Simultaneously, this increase of SINR allows 100% coverage probability at high altitudes: up to 50 m for the standard three-sector per BS case, reaching up to 100 m for a case with 42–45 sectors per BS using mmWave.

We demonstrated that a significant improvement of the coverage of AUEs at high altitudes is possible using mmWave, even when only a basic form of beamforming is applied. We can expect that the research community will concentrate its future work at mmWave and beamforming techniques when exploring ways to provide coverage for high-altitude UAVs. It is a promising direction towards the 3D cellular networks for urban environments. 

## Figures and Tables

**Table 1:** Simulation parameters.

Parameters	Values
Carrier frequencies	1.8 GHz, 38 GHz
Signal bandwidth, B	20 MHz, 400 MHz
White noise power density, $N_{0}$ [20]	−174 dBm/Hz
UE noise figure, F [20]	9 dB
Active ground user density, ρ	20 users/km${}^{2}$
# iterations	100

**Table 2:** Antenna parameters for different number of sectors per base station.

# Sectors per BS	3	6	9	12	15	18	21	24	27	30	33	36	39	42	45
$\phi_{3}\;{[}^{\circ }]$	65	32.5	21.67	16.25	13	10.83	9.29	8.13	7.22	6.5	5.91	5.42	5	4.64	4.33
$G_{0}\;\left[dBi\right]$	18	21.01	22.77	24.02	24.99	25.78	26.45	27.03	27.54	28	28.41	28.79	29.14	29.46	29.76
$P_{tx}\;\left[dBm\right]$	46	42.99	41.23	39.98	39.01	38.22	37.55	36.97	36.46	36	35.59	35.21	34.86	34.54	34.24

## Abbreviations

The following abbreviations are used in this manuscript:

AGL	Above Ground Level
AUE	Aerial User Equipment
BS	Base Stations
LOS	Line-of-Sight
LTE	Long-Term Evolution
MaMIMO	Massive Multiple Input Multiple Output
mmWave	Millimeter wave
NLOS	Non-line-of-Sight
PL	Path loss
SINR	Signal-to-Interference-plus-Noise Ratio
UAV	Unmanned Aerial Vehicles
UE	User Equipment
